# Author Correction: Olivine-rich achondrites from Vesta and the missing mantle problem

**DOI:** 10.1038/s41467-021-26515-1

**Published:** 2021-10-21

**Authors:** Zoltan Vaci, James M. D. Day, Marine Paquet, Karen Ziegler, Qing-Zhu Yin, Supratim Dey, Audrey Miller, Carl Agee, Rainer Bartoschewitz, Andreas Pack

**Affiliations:** 1 Institute of Meteoritics, University of New Mexico, Albuquerque, NM USA; 2 Department of Earth and Planetary Sciences, University of New Mexico, Albuquerque, NM USA; 3 Scripps Institution of Oceanography, University of California San Diego, La Jolla, CA USA; 4 Department of Earth and Planetary Sciences, University of California Davis, Davis, CA USA; 5 Bartoschewitz Meteorite Laboratory, Gifhorn, Germany; 6 Georg-August-Universität, Göttingen, Germany

**Keywords:** Geochemistry, Meteoritics, Petrology

Correction to: *Nature Communications* 10.1038/s41467-021-25808-9, published online 14 September 2021.

The original version of this Article contained an error in Fig. 2, in which outdated Cr isotopic data has been reported. This has been corrected in both the PDF and HTML versions of the Article.

