# Supplementary material for: Olivine-rich achondrites from Vesta and the missing mantle problem
Source: Nat Commun. 2021 Sep 14;12:5443. doi: 10.1038/s41467-021-25808-9 (PMC8440560; doi:10.1038/s41467-021-25808-9)
Supplement: Supplementary file 1 — Supplementary Information [file 41467_2021_25808_MOESM1_ESM.pdf]

Supplementary Information

Sample deposit information

Northwest Africa 12217 was purchased by Jay Piatek in 2015 from a Moroccan meteorite dealer, and a 20.7 g deposit sample resides at the University of New Mexico (UNM). Northwest Africa 12319 was discovered in 2018 as thirteen fragments and splinters totaling 35.3 g, and it was sold by a meteorite dealer in Agadir, Morocco, to Alan Mazur in July 2018. Deposit samples reside at the Universities of Kiel (5.07 g) and Münster (1.05 g). Northwest Africa 12562 was found in Algeria in 2017 as a single 3930 g stone, and it was purchased by Zuokai Ke in May 2018 in Chenzhou, China. A 21 g deposit sample resides at the University of New Mexico.

Supplementary Discussion

The possibility of subsolidus introduction of exogenous chromium to planetary materials warrants discussion. The symplectites identified in NWA 12217, 12319, and 12562 are commonly found in ultramafic and mafic rocks, including examples within terrestrial peridotites, lunar troctolites, martian basalts and cumulates, diogenites, and the ungrouped achondrite QUE 93148. The particular type of symplectites found in NWA 12217, 12319, and 12562 are almost identical to those found in the Mg-rich harzburgite clasts in howardites[1]. They are composed of vermicular chromite lathes, low-Ca pyroxene, and high-Ca pyroxene (Fig. S11 and S12). The pyroxene included in the symplectites is generally more magnesian (FeO < 2.0 wt.%) than pyroxenes not associated with symplectites. The petrogenesis of symplectites is controversial, with many possible formation mechanisms proposed over decades of research[1–9]. These include: (1) crystallization of late-stage trapped melts; (2) diffusion of minor elements out of olivine; (3)

breakdown of pre-existing chrome garnets; (4) reaction with a chromium-rich metasomatizing fluid.

It is unlikely that the symplectites are late-stage crystallization products because their bulk composition is not that of a possible melt inclusion or late-stage residuum[5]. The amount of chromium dissolved in the melt would have to be unreasonably high to crystallize such phases. A likely candidate for such a late-stage melt pocket was identified in NWA 12217. The bulk composition of the pocket is much higher in Si, Al, Ca, than the symplectites and contains negligible amounts of Cr. Similar to the symplectites in a lunar troctolite[7], the symplectites contain highly variable proportions of high- and low-Ca pyroxene, but the compositions of these phases are largely uniform. The composition of a trapped melt would be highly variable depending on the stage of the crystallization sequence of the parent magma, and this variability should be reflected in pyroxene compositional diversity. Conversely, if each trapped pocket was formed at the same time, a uniform distribution of high- and low-Ca pyroxene should be expected. We find neither to be the case.

We also consider it to be unlikely that the symplectites were formed by diffusion of Cr out of olivine. The Cr contents of olivines in all three meteorites are uniformly low (Fig. S7), and there are chromite grains scattered throughout that were likely liquidus phases and the primary Cr carriers. None of the olivine grains contain diffusion gradients in Cr or any other element, which would be expected if Cr was diffusing out of the grains and into the symplectites. Instead, there are elevated Cr compositions in olivines adjacent to the symplectites which implies Cr diffusion from the symplectites into the olivines, as found in the lunar troctolite[7] and in Vestal Mg-rich harzburgites[1]. We note that the elevated Cr in olivine grains is only observed one or two

μm away from the symplectites, so it is also possible that the measurement is due to symplectite interference with the electron beam's interaction volume during microprobe analysis.

The bulk compositions of the symplectites are stoichiometrically consistent with having been formed through the breakdown of preexisting chromium garnet[9]. However, the garnet stability field exists at pressures greater than 1.5 GPa[10], a regime difficult to reconcile with formation on a small body such as a planetesimal. Even at the high end of a theoretical pressure calculation, assuming a body with a diameter of 1000 km, an average density of 4000 kg/m^3, and a depth of 400 km, the hydrostatic pressure is less than 1 GPa. We suggest that NWA 12217, 12319, and 12562 more likely formed on a smaller body, and therefore the symplectites are unlikely to be garnet breakdown products. However, as the planetesimal size is poorly constrained, this possibility cannot be completely excluded.

There is also the possibility of a Cr-rich metasomatizing melt or fluid crystallizing symplectites like those identified in NWA 12217, 12319, and 12562. Elardo et al. (2012) suggested that a chromite-rich basaltic melt infiltrated cracks and grain boundaries in the lunar troctolite 76535 and crystallized chromite as a liquidus phase. The chromite then reacted metasomatically with bounding olivine and plagioclase, resulting in the formation of symplectites with high Mg# since they were buffered by the high Mg# of adjacent olivine. Additional unreacted chromite was deposited as chromite veins. Hahn et al. (2018) likewise argued for an exogenous origin for the symplectites they identified in Mg-rich harzburgite howardite clasts. They found a glassy melt inclusion high in Cr with a preserved reaction front that was in the process of precipitating a symplectite assemblage. Based on this observation, they argued that a melt that contained both a silicate and metallic component interacted with olivine under changing fO_2 conditions and resulted in the crystallization of the symplectites.

Based on our observations, we do not consider that the mechanisms proposed by Elardo et al. (2012) to be relevant to symplectite formation in NWA 12217, 12319, or 12562, since the symplectites are ubiquitous as inclusions within olivine and along olivine-olivine and olivine-pyroxene grain boundaries and do not involved plagioclase. Hahn et al. (2018) present an interesting model of formation based on a single melt glass that they identified interacting with a symplectite. However, we found no such melt in NWA 12217, 12319 or 12562. Therefore, we do not find it compelling to associate these symplectites with a hypothetical infiltrating melt. Furthermore, the melt glass[1] remains poorly characterized, and likely finer-scale measurements such as TEM analyses will be necessary to understand how such a melt could accommodate high levels of Cr_2O_3, along with the microstructure of the propagating reaction front. Their suggestion of a metallic melt component necessary to incorporate Cr and an associated increase in fO_2 also remains hypothetical.

The main obstacle to deciphering the origin of the symplectites in NWA 12217, 12319, and 12562 is the origin of chromium. The most obvious source would be the chromite that is ubiquitous throughout all three meteorites, but implicating these requires that they break down spontaneously into symplectites in some regions while remaining pristine in others, with no satisfying explanation as to why. There is also no clear source for the excess silica required to crystallize the pyroxene components of the symplectites. In lieu of any other plausible sources for Cr, we find that the interaction of an exogenous melt is a possible formation mechanism, but there is a paucity of evidence for such an interaction.

Supplementary Figures

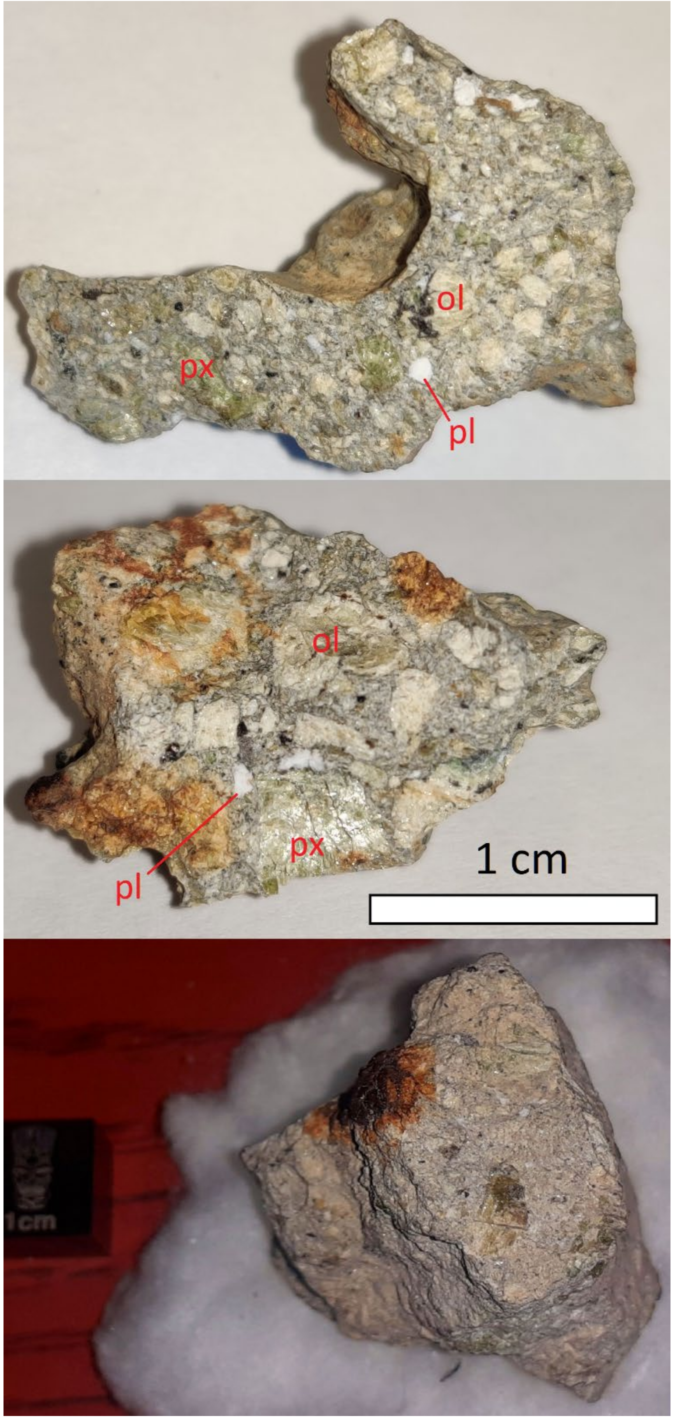

Figure S1. Hand-sample images of NWA 12562 (top two images) with major phases labeled (ol = olivine; px = pyroxene; pl = plagioclase) and NWA 12319 (bottom). Black grains are chromites and chromium symplectites. Brown areas are stained by terrestrial weathering.

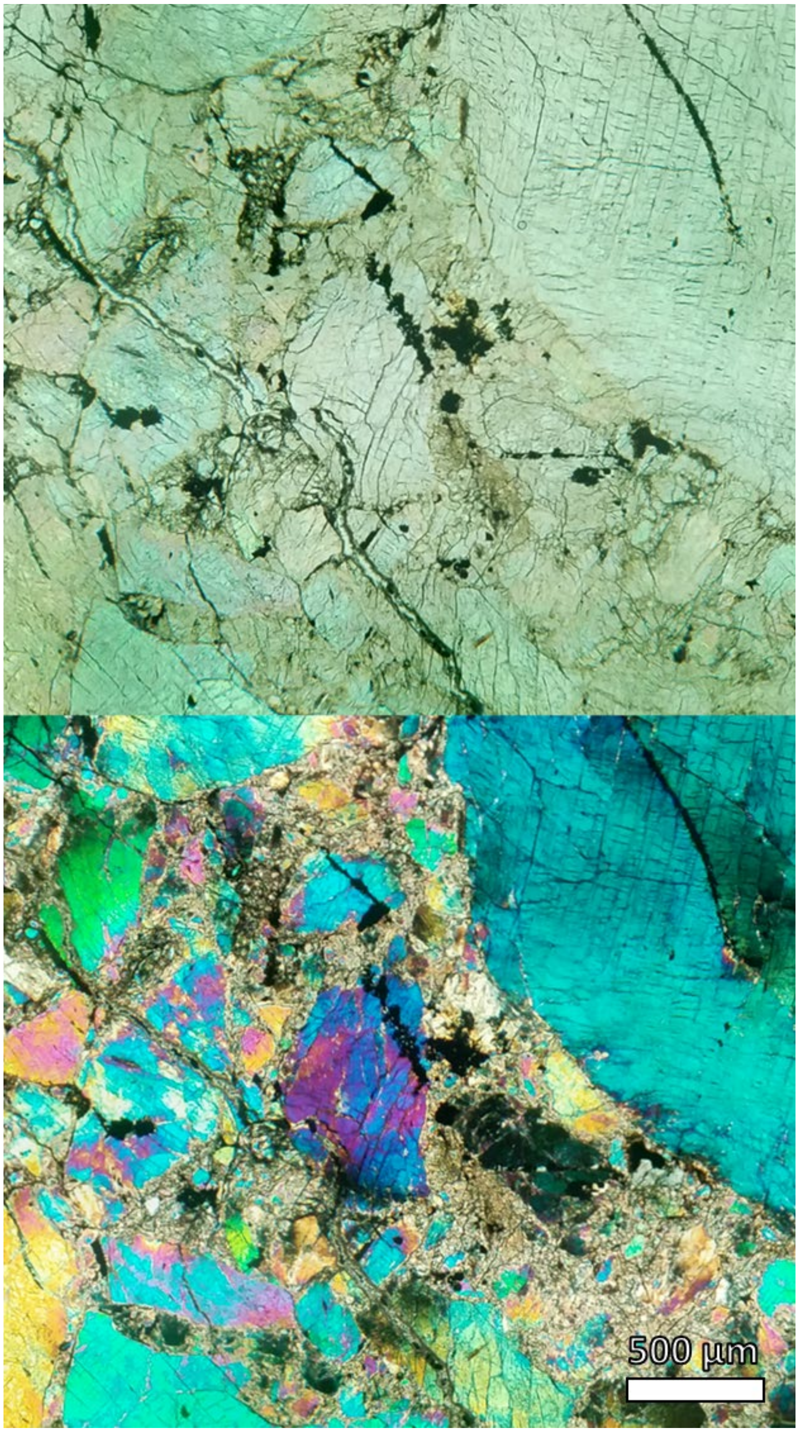

Figure S2. Plane-polarized light (PPL) (top) and cross-polarized light (XPL) (bottom) images of the same area in NWA 12217, showing undulose extinction, fracturing, and shock-mosaicism of compositionally homogenous olivine.

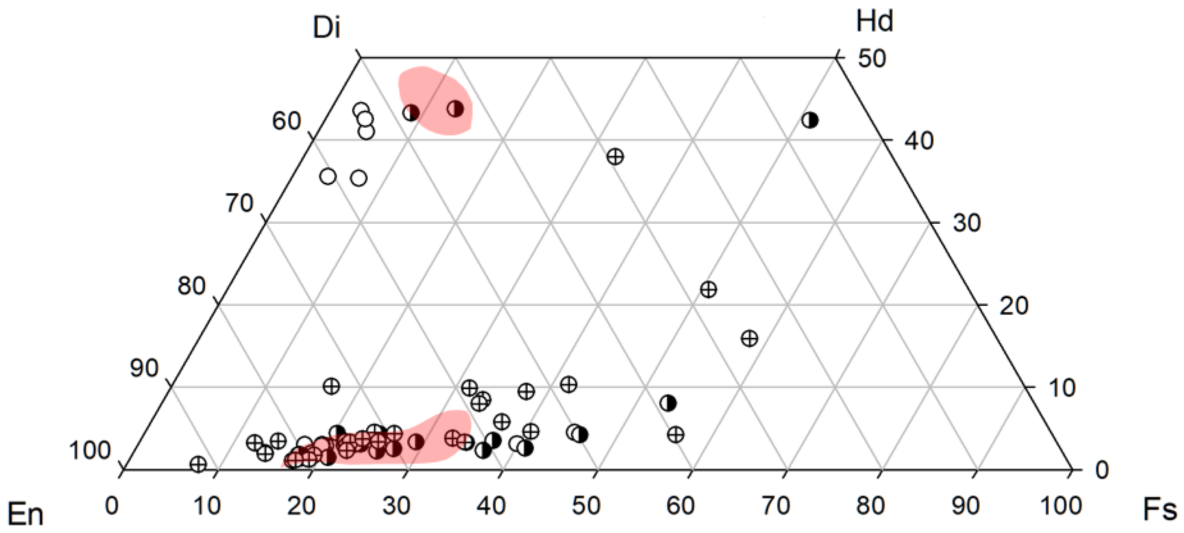

Figure S3. Quadrilateral showing pyroxene compositions of dunite NWA 12217 (open circles), lherzolites NWA 12319 (half-filled circles) and 12562 (crossed circles), and fields of diogenites[11] (pale red).

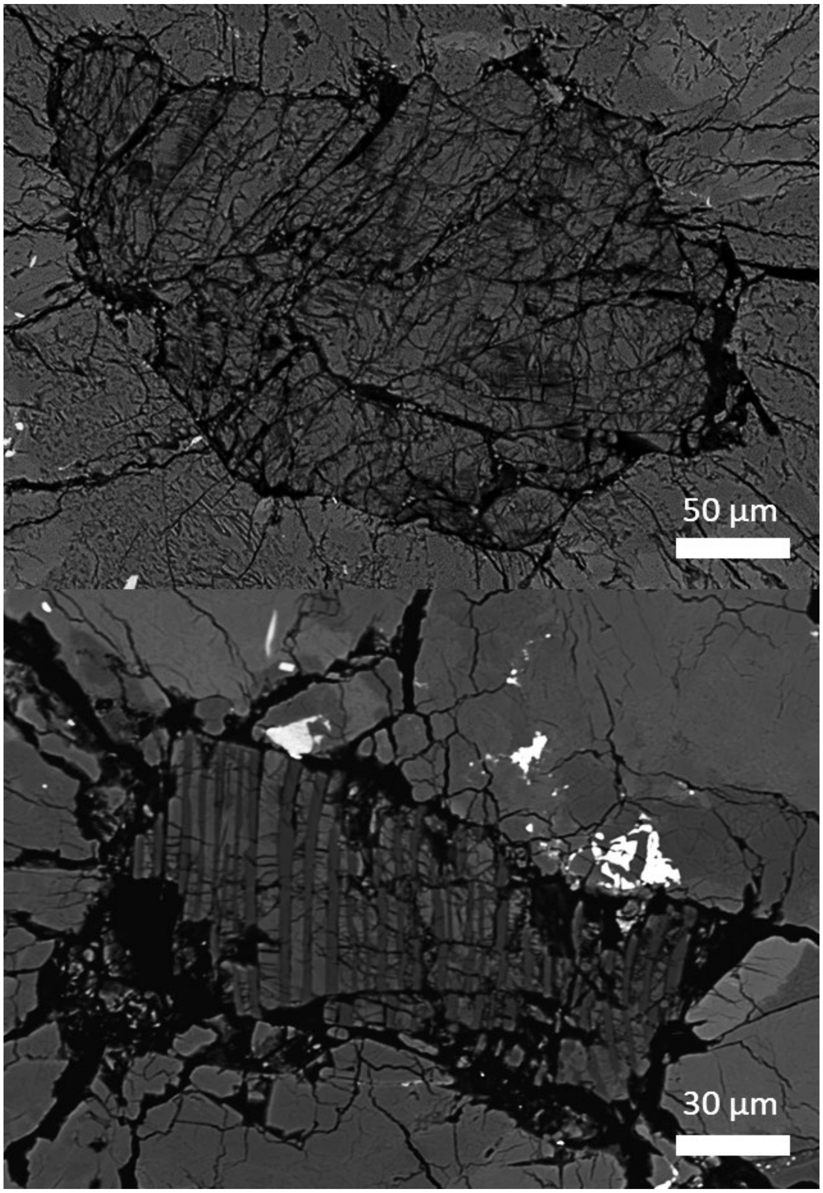

Figure S4. BSE images of plagioclase (top) and alternating lathes of K-feldspar and silica (bottom), both found as inclusions within olivine grains in NWA 12217.

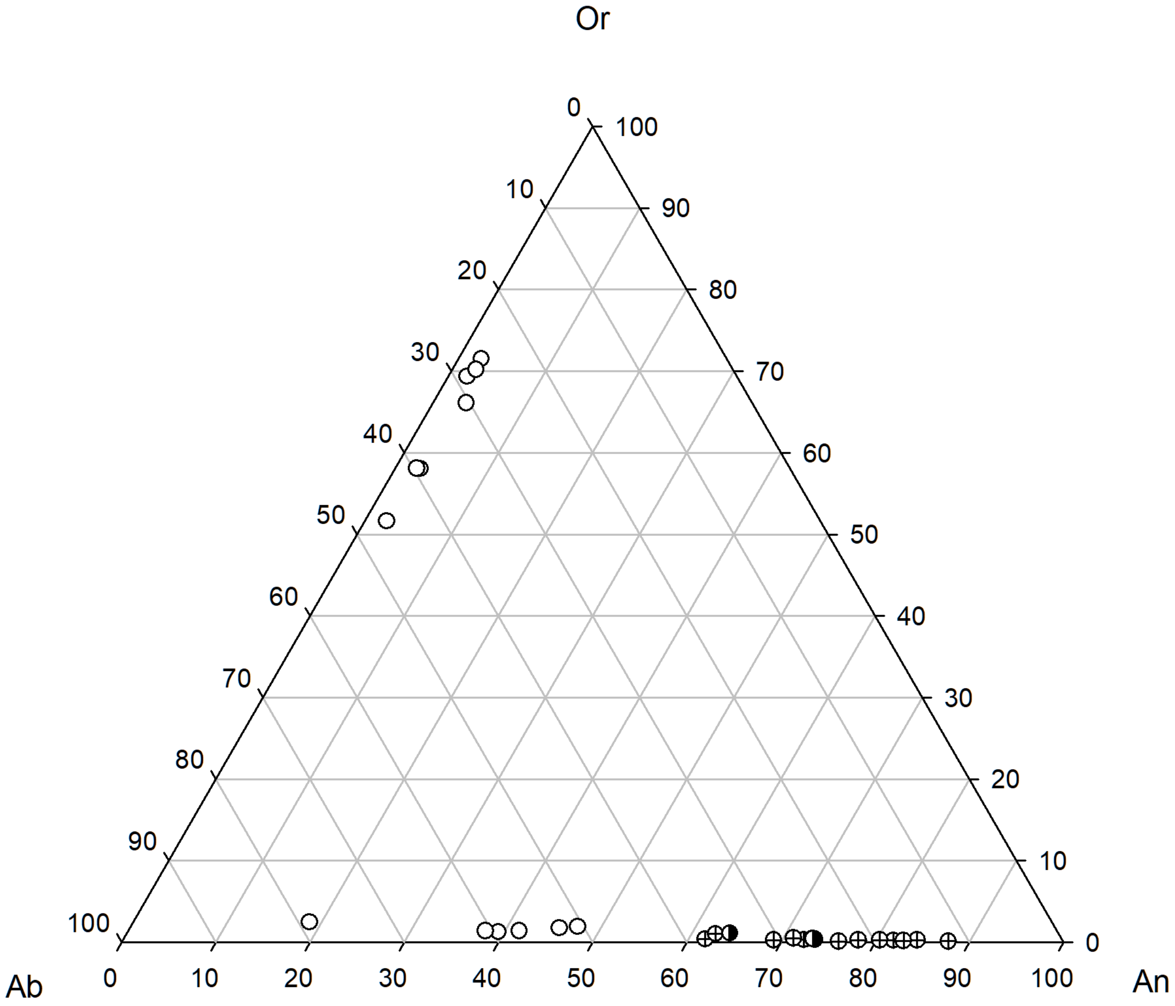

Figure S5. Ternary diagram showing feldspar compositions in NWA 12217 (open circles), 12319 (half-filled circles), and 12562 (crossed circles). Feldspars in NWA 12217 are small (<500 μm) inclusions in olivine and are likely representative of trapped melt, while feldspars in NWA 12319 and 12562 appear as brecciated clasts.

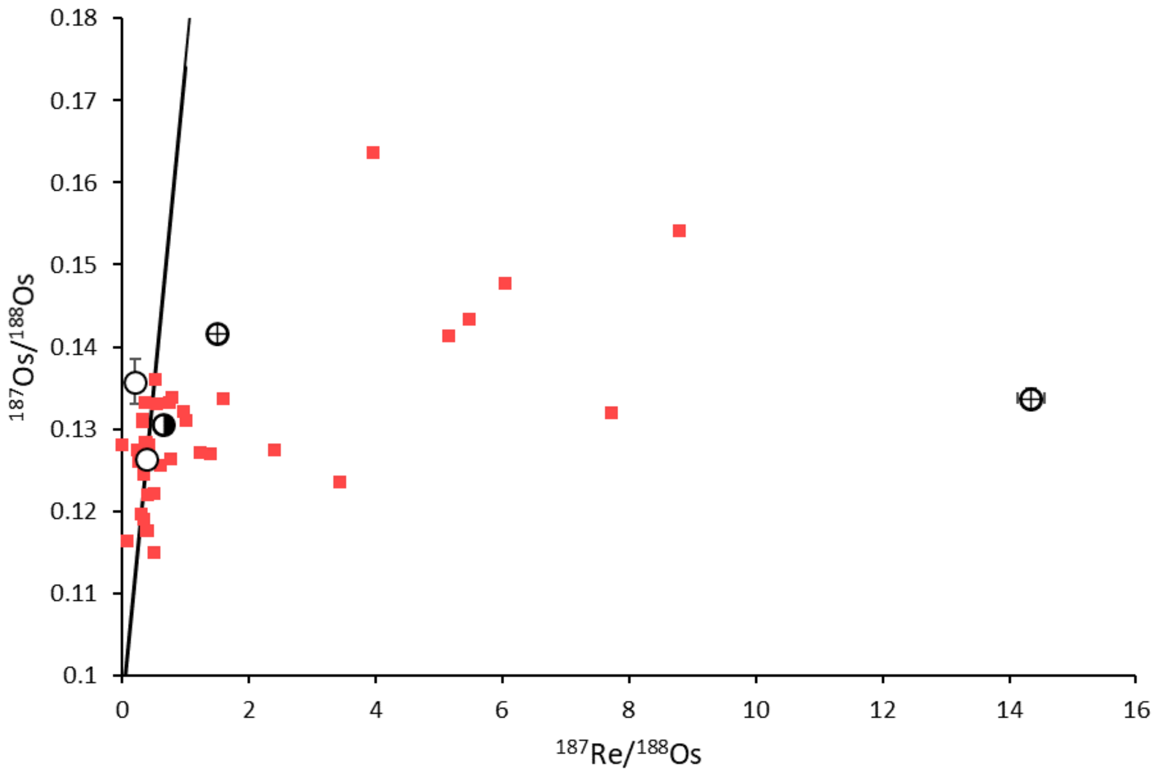

Figure S6. Rhenium-Os Isochron plot showing NWA 12217 (open circles), NWA 12319 (half filled circle), NWA 12562 (crossed circles), HED meteorites[12,13] (red), and the 4.568 Ga IIIAB iron meteorite isochron[14].

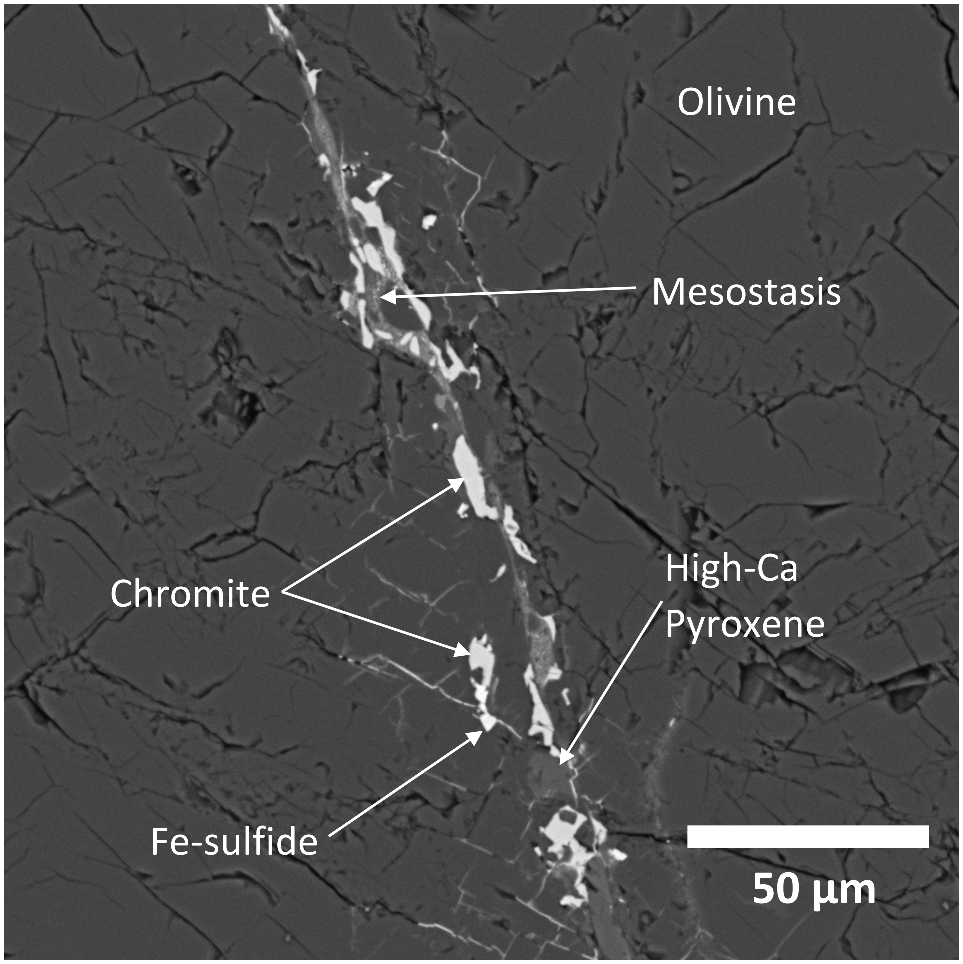

Figure S7. Vein-like feature ubiquitous in NWA 12217 and 12562. Veins are found within olivine grains and along grain boundaries, and some are disturbed by shock. Symplectites (Fig. S1) are sometimes found within veins.

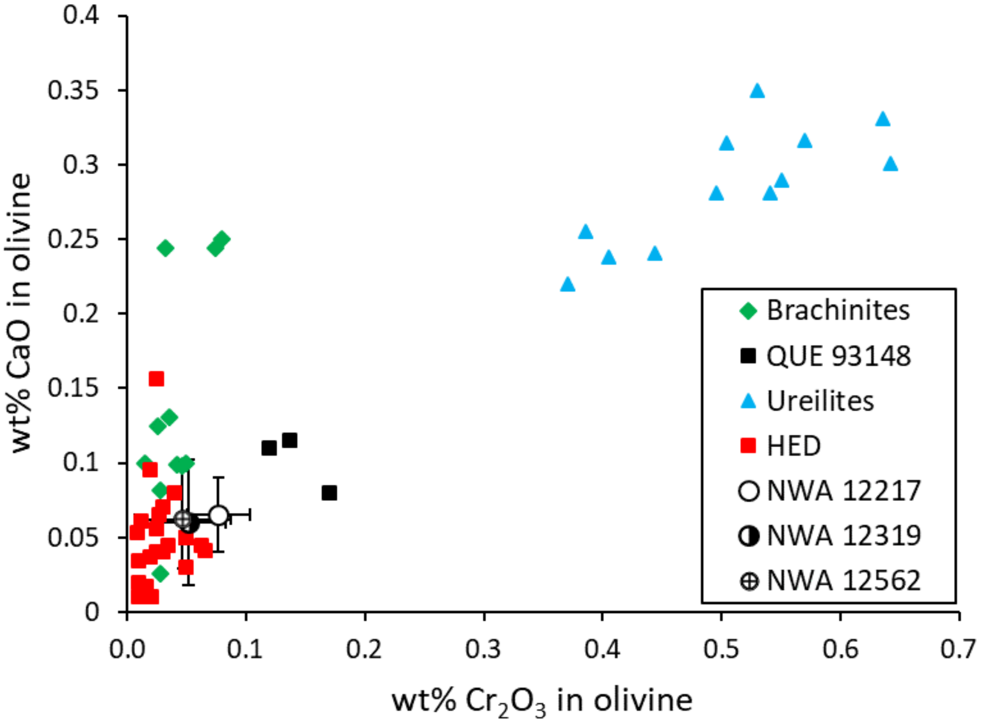

Figure S8. Average CaO versus Cr_2O_3 content in olivine grains in NWA 12217, 12319, and 12562, plotted with olivines from other meteorite groups and QUE 93148[11,15–18].

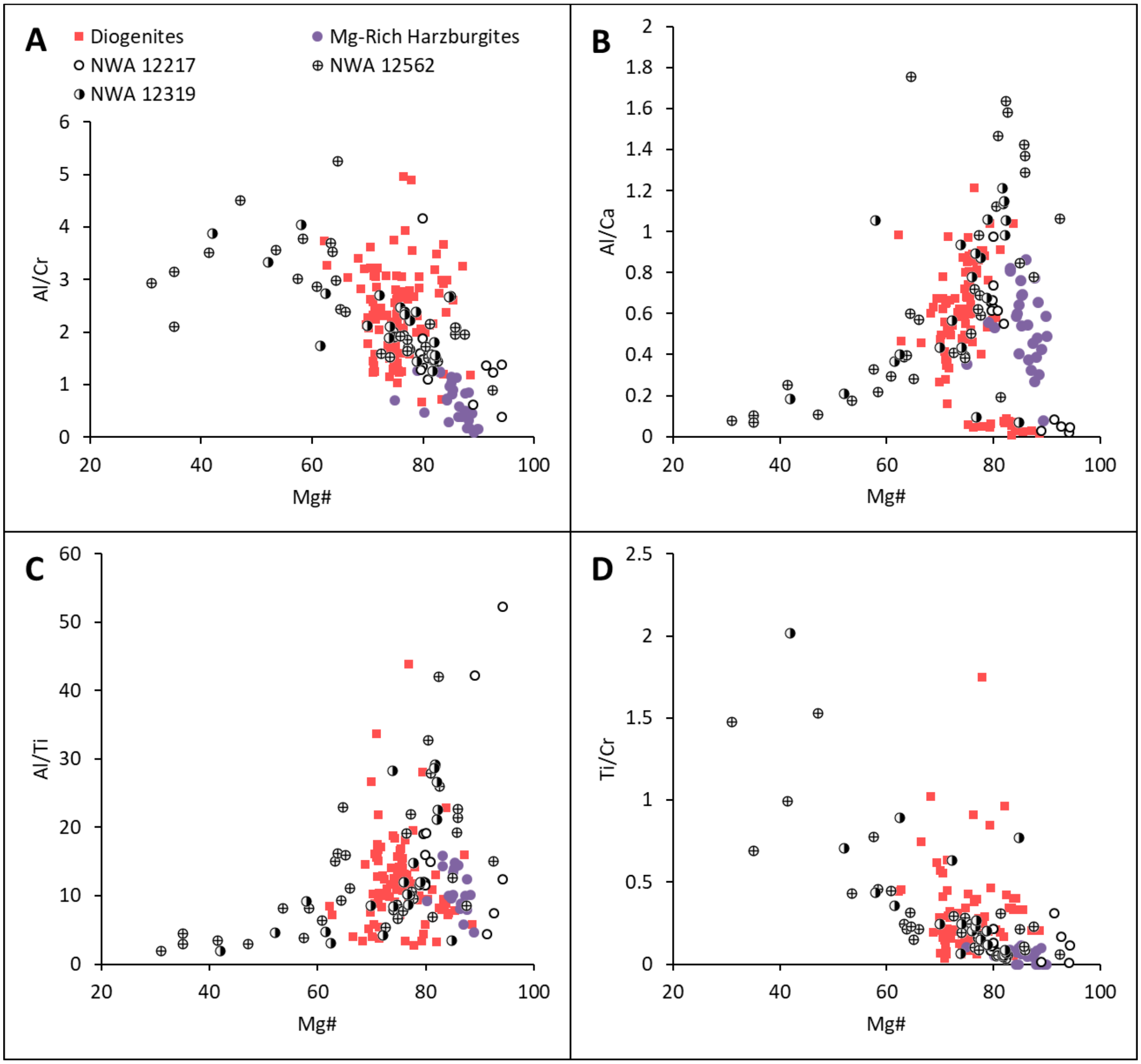

Figure S9. Plots of Mg# versus (a) Al/Cr, (b) Al/Ca, (c) Al/Ti and (d) Ti/Cr in pyroxenes. NWA 12217 and 12562 form largely continuous trends with the Mg-rich harzburgites[1] and diogenites[11] in all data sets except for Al/Ca, in which the Mg-rich harzburgites show a separate trend.

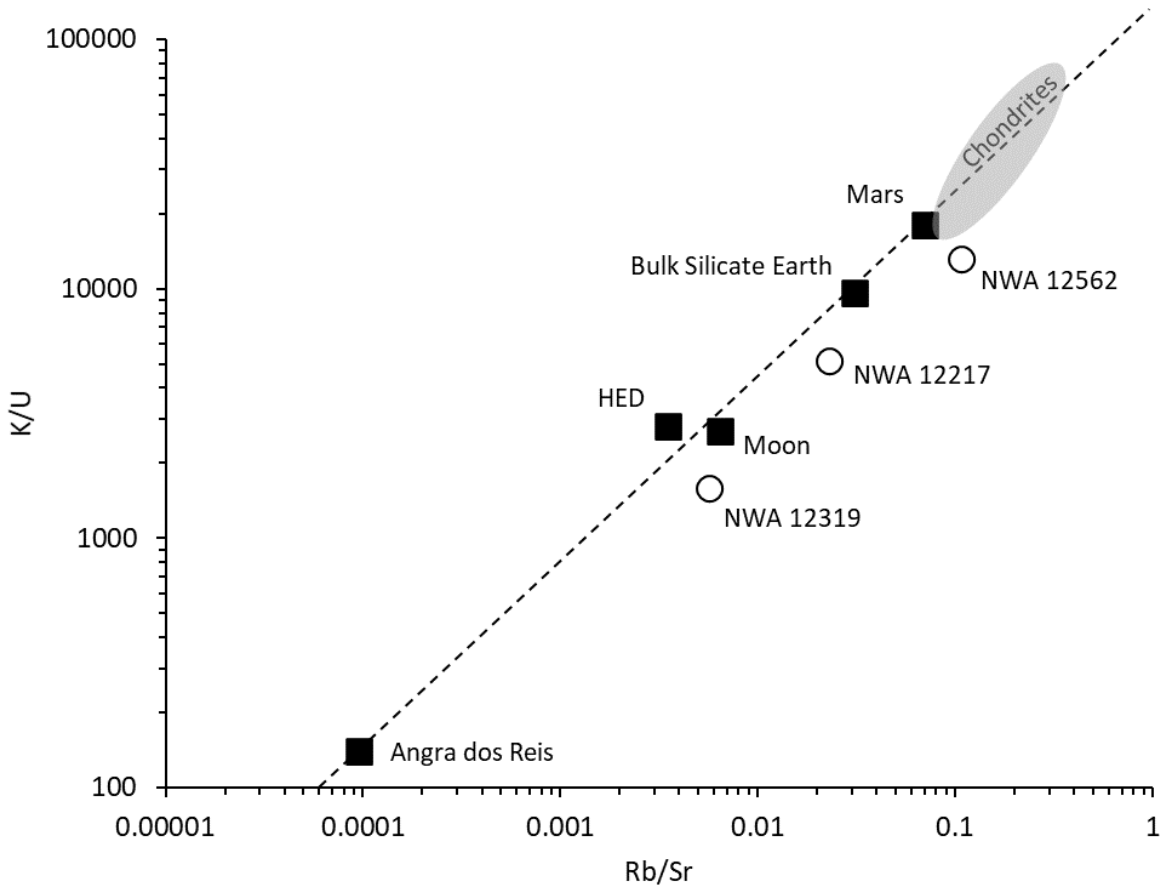

Figure S10. Potassium/U versus Rb/Sr as a proxy for planetary volatile depletion[19]. The ultramafic achondrites plot off of the trend formed by the rest of the solar system and show extreme variation, suggesting that they are not representative of their parent bodies. In addition to modification by terrestrial weathering, these elements are subject to 'nugget effects' by brecciation and sample aliquot selection, especially in these olivine-dominated rocks.

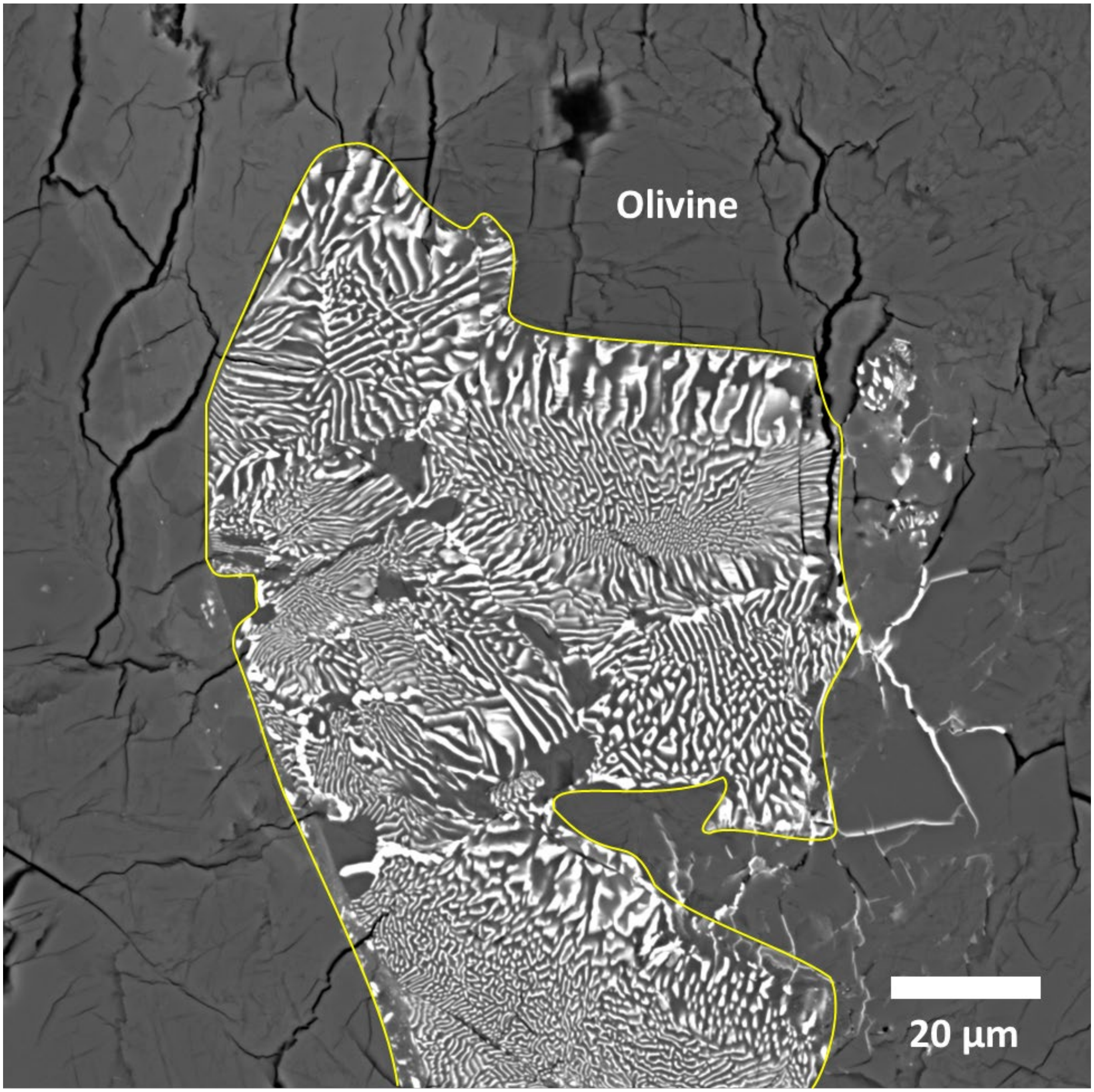

Figure S11. Backscatter electron (BSE) image of a symplectite (yellow outline) composed of chromite and low- and high-Ca pyroxene, completely enclosed within an olivine grain.

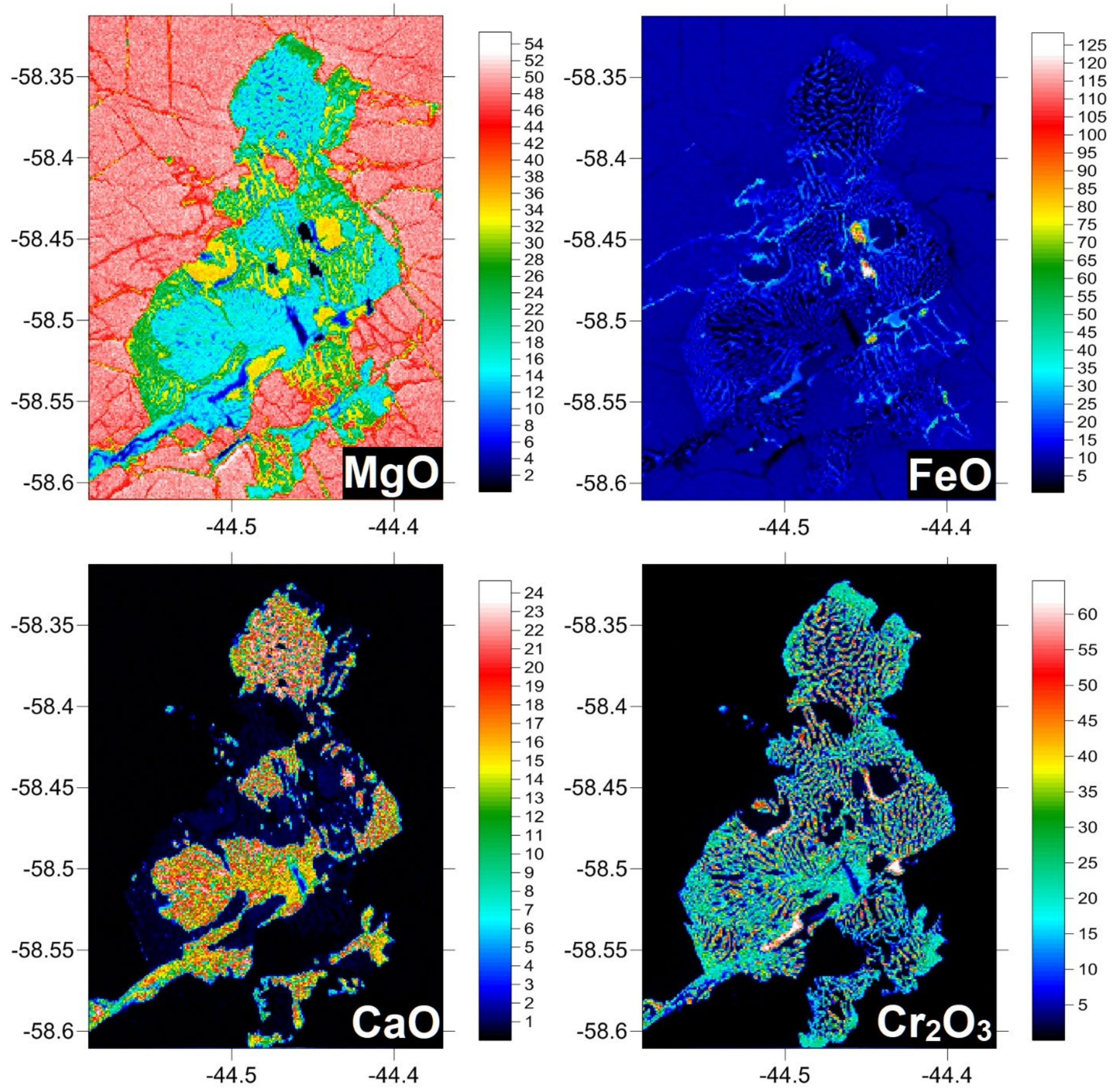

Figure S12. Quantitative X-ray oxide maps of a symplectite in NWA 12217. High FeO spots are FeNi metal or Fe-sulfide grains. Dimensions are in mm; color scale is in wt.%.

References

1. Hahn, T. M., Lunning, N. G., McSween, H. Y., Bodnar, R. J. & Taylor, L. A. Mg-rich harzburgites from Vesta: Mantle residua or cumulates from planetary differentiation? *Meteorit. Planet. Sci.* **53**, 514–546 (2018).
2. Bell, P. M., Mao, H. K., Roedder, E. & Weiblen, P. W. The problem of the origin of symplectites in olivine-bearing lunar rocks. *Proc. lunar Sci. Conf. 6th* 231–248 (1975) doi:10.1017/CBO9781107415324.004.
3. Dawson, J. B. & Smith, J. V. Chromite-silicate intergrowths in upper-mantle peridotites. *Phys. Chem. Earth* **9**, 339–350 (1975).
4. Field, S. W. Diffusion, discontinuous precipitation, metamorphism, and metasomatism: The complex history of South African upper-mantle symplectites. *Am. Mineral.* **93**, 618–631 (2008).
5. Holness, M. B. *et al.* Silicate liquid immiscibility within the crystal mush: late-stage magmatic microstructures in the skaergaard intrusion, east greenland. *J. Petrol.* **52**, 175–222 (2011).
6. Morishita, T. & Arai, S. Evolution of spinel-pyroxene symplectite in spinel-lherzolites from the Horoman Complex, Japan. *Contrib. to Mineral. Petrol.* **144**, 509–522 (2003).
7. Elardo, S. M., McCubbin, F. M. & Shearer, C. K. Chromite symplectites in Mg-suite troctolite 76535 as evidence for infiltration metasomatism of a lunar layered intrusion. *Geochim. Cosmochim. Acta* **87**, 154–177 (2012).
8. Khisina, N. R., Wirth, R., Abart, R., Rhede, D. & Heinrich, W. Oriented chromite-diopside symplectic inclusions in olivine from lunar regolith delivered by ‘ Luna-24’ mission. *Geochim. Cosmochim. Acta* **104**, 84–98 (2013).
9. Špaček, P., Ackerman, L., Habler, G., Abart, R. & Ulrych, J. Garnet breakdown, symplectite formation and melting in basanite-hosted peridotite xenoliths from zinst (bavaria, bohemian massif). *J. Petrol.* **54**, 1691–1723 (2013).
10. O’Hara, M. J., Richardson, S. W. & Wilson, G. Garnet-peridotite stability and occurrence in crust and mantle. *Contrib. to Mineral. Petrol.* **32**, 48–68 (1971).
11. Mittlefehldt, D. W. Asteroid (4) Vesta: I. The howardite-eucrite-diogenite (HED) clan of meteorites. *Chemie der Erde - Geochemistry* vol. 75 155–183 (2015).
12. Dale, C. W. *et al.* Late accretion on the earliest planetesimals revealed by the highly siderophile elements. *Science* **335**, 72–75 (2012).
13. Day, J. M. D., Walker, R. J., Qin, L. & Rumble, D. Late accretion as a natural consequence of planetary growth. *Nat. Geosci.* **5**, 614–617 (2012).
14. Smoliar, M. I., Walker, R. J. & Morgan, J. W. Re-Os ages of group IIA, IIIA, IVA, and IVB iron meteorites. *Science* **271**, 1099–1102 (1996).
15. Goodrich, C. A., Fioretti, A. M., Tribaudino, M. & Molin, G. Primary trapped melt inclusions in olivine in the olivine-augite-orthopyroxene ureilite Hughes 009. *Geochim. Cosmochim. Acta* **65**, 621–652 (2001).
16. Goodrich, C. A., Wlotzka, F., Ross, D. K. & Bartoschewitz, R. Northwest Africa 1500: Plagioclase-bearing monomict ureilite or ungrouped achondrite? *Meteorit. Planet. Sci.* **41**, 925–952 (2006).
17. Goodrich, C. A. & Righter, K. Petrology of unique achondrite Queen Alexandra Range 93148: A piece of the pallasite (howardite-eucrite-diogenite?) parent body? *Meteorit. Planet. Sci.* **35**, 521–535 (2000).
18. Mittlefehldt, D. W., Bogard, D. D., Berkley, J. L. & Garrison, D. H. Brachinites: Igneous

rocks from a differentiated asteroid. *Meteorit. Planet. Sci.* **38**, 1601–1625 (2003).
19. Halliday, A. N. & Porcelli, D. In search of lost planets - The paleocosmochemistry of the inner solar system. *Earth Planet. Sci. Lett.* **192**, 545–559 (2001).
