# Supplementary material for: Olivine-rich achondrites from Vesta and the missing mantle problem
Source: Nat Commun. 2021 Sep 14;12:5443. doi: 10.1038/s41467-021-25808-9 (PMC8440560; doi:10.1038/s41467-021-25808-9)
Supplement: Supplementary file 3 — Description of Additional Supplementary Files [file 41467_2021_25808_MOESM3_ESM.pdf]

Description of Additional Supplementary Files

File name: Supplementary Data 1
Description: Microprobe data, major and trace element analyses, highly siderophile element analyses, and isotopic analyses for ultramafic achondrites.
